# Electrode- and Label-Free Assessment of Electrophysiological Firing Rates through Cytochrome C Monitoring via Raman Spectroscopy

**DOI:** 10.1021/acssensors.4c03133

**Published:** 2025-02-05

**Authors:** Christian Tentellino, Marta d’Amora, Rustamzhon Melikov, Giuseppina Iachetta, Giulia Bruno, Francesco Tantussi, Michele Dipalo, Francesco De Angelis

**Affiliations:** 1 Plasmon Nanotechnologies, Istituto Italiano di Tecnologia, Via Morego 30, Genoa 16153, Italy; 2 Department of Biology, University of Pisa, S.S. 12 Abetone e Brennero, 4, Pisa 56127, Italy

**Keywords:** firing rate, cytochrome C, Raman spectroscopy, electrophysiology, neurotoxicity, multielectrode array

## Abstract

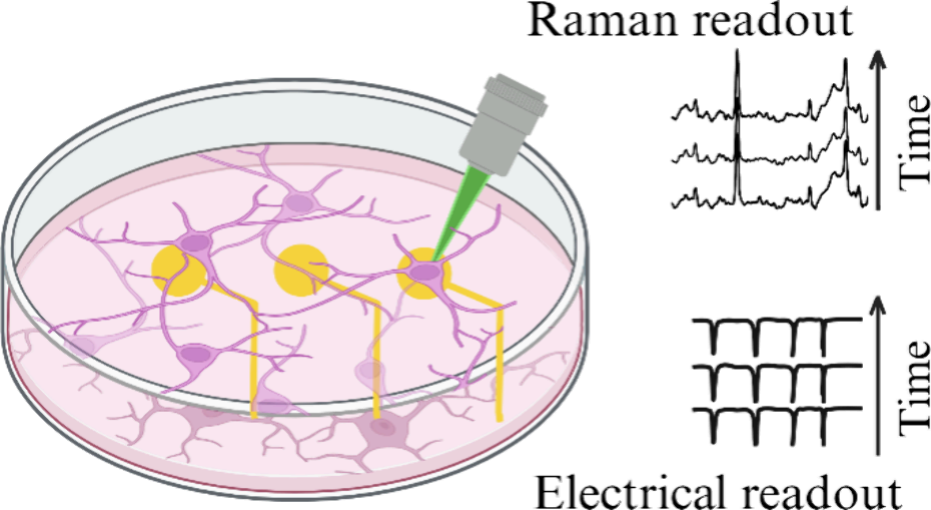

*In vitro* neurotoxicology aims to assess and predict the side effects of exogenous chemicals toward the human brain. Among the exploited approaches, electrophysiological techniques stand out for the high spatiotemporal resolution and sensitivity, with the patch clamp considered the gold standard technique for such purposes. However, structural toxicity and metabolic effects may elude detection when only the electrical activity is measured, highlighting the need for integrating electrophysiological recordings with complementary approaches such as optical methods. In this study, we describe an integrated platform for recording neuronal electrical activity and performing chemical analysis with a noninvasive label-free optical imaging, Raman spectroscopy. Specifically, we developed a protocol that maximizes the signal-to-noise ratio while avoiding the crosstalk of the electrical and spectroscopical readouts and any phototoxicity associated with the laser exposure. Synchronous and sequential electrical–optical measurements were carried out and compared, with the sequential approach being more suitable for the longitudinal investigation and correlation of the neuronal electrical activity to the intracellular content of reduced cytochrome C, lipids, proteins, and nucleic acids. Data analysis shows a strong correlation between the metabolic status of the single cells and the overall neuronal firing rate, suggesting the electrode- and label-free assessment of the neuronal firing rates through the monitoring of cytochrome C via Raman spectroscopy when multielectrode array devices with high electrical noise and impedance are used. Conversely, the neuronal firing rate and the reduced cytochrome C content were not correlated to lipids, proteins, and nucleic acids. Thus, this study demonstrates the crosstalk of the neuronal firing rate and reduced cytochrome C as downstream and upstream features of the neuronal metabolic activity and that through the monitoring of the *de novo* synthesis of lipids, proteins, and nucleic acids, Raman spectroscopy provides additional information for a more accurate assessment of the acute and chronic neurotoxicity.

*In vitro* neurotoxicology aims to screen and examine the activity of exogenous compounds toward the neuronal activity following acute and chronic exposure. To do so, neurotoxicologists require the (i) development of representative 2D–3D neuronal-like cultures and (ii) the development of technologies that enable to integrate and correlate functional and structural features with metabolic and molecular changes occurring at the subcellular level.

Up to date, the development of self-assembling brain spheroids and organoids based on animal and human-induced pluripotent stem cells recalling the complex cytoarchitecture of the brain has been largely used to unveil neurotoxicity.^1^ This is exemplified by Nzou *et al*., who successfully reproduced the blood–brain barrier on a human cortex spheroid,^1f^ and Takayama *et al*., who successfully reproduced an *in vitro* autonomic nervous system, enabling the control over the cardiomyocytes’ beating through the stimulation of sympathetic and parasympathetic-like neurons.^2^

While neuronal models are advancing very fast, commercially available techniques for the investigation of neurotoxicity still suffer from significant limitations. In this regard, the development of methods providing a nondestructive, longitudinal readout of neuronal biochemical changes occurring in physiological and cytotoxic events would be extremely beneficial to increase the reliability and accuracy.

In neurotoxicology, drug toxicity evaluations commonly exploit a variety of electrical and optical measurements,^3^ with patch clamp^4^ and fluorescence microscopy^5^ being the gold standard techniques in each field. However, these readouts are perturbative to the biological samples, unable to carry out longitudinal measurements, and usually carried out separately. Thus, their predictive power is reduced while increasing time and costs. With these premises, the development of a longitudinal, synergically working multivariable investigation tool enabling the correlation of different biological features in response to drug treatment would represent a pioneering technology and would likely enable unveiling previously “hidden” neurotoxicity. Clearly, it does require the use of methods that are nondestructive and with minimal or absent perturbation of physiological conditions. Microelectrode array (MEA) devices enable the noninvasive long-term electrical readout of cell networks with a high spatiotemporal resolution,^6^ with the major drawback represented by the high electrical noise and impedance when small electrodes’ sizes (diameter = <5 μM) are used.^7^

Similarly, Raman spectroscopy is a label-free optical imaging technique that offers a readout on cellular phenotypes, metabolic pathways, and cellular stress down to the molecular level.^8^ Hence, the combination of Raman spectroscopy with MEA recordings would provide a formidable tool for the investigation of neuronal cultures by integrating real-time and label-free electrical readouts with biomolecular insights, enabling the simultaneous, real-time monitoring of the biochemical responses and the changes in neuronal activity. Raman spectroscopy can also monitor the dynamics of cytochrome C, an endogenous biomolecule that contributes to the oxidative phosphorylation (OxPhos) and the synthesis of adenosine triphosphate (ATP), the fuel source of the cells, acting as an electron shuttle between the third and fourth complex of the electron transport chain (ETC). Of note, cytochrome C oxidase, the fourth enzymatic complex of the ETC, is a well-established biomarker of neuronal health.^9^ Accordingly, the neuronal electrical activity, an electrophysiological feature associated with healthy neuronal cultures, is strictly dependent from OxPhos and allosterically modulates the enzymatic activity of the cytochrome C oxidase, the rate-limiting enzyme of the ETC,^10^ thus suggesting that an increased content of the reduced cytochrome C may reflect the overall neuronal activity and represents a biomarker for following the neuronal activity and health status.

Up to date, the implementation of electrophysiological means and Raman spectroscopy for the assessment of neuronal cultures has been hampered by technical challenges such as (i) the overwhelming fluorescent biological medium that interferes with the collection of the Raman scattering originating from the sample following its exposure to visible light; notably, the overall Raman scattering cross section is about 10^–30^ cm^2^ per chemical bond, indicating how a minimum level of fluorescence can easily obscure Raman scattering;^11^ (ii) the fourth power relationship between the wavelength of excitation and the Raman scattering, “forcing” the use of visible sources rather than NIR ones for the extraction of Raman features with a high signal-to-noise ratio;^12^ (iii) the phototoxicity mediated by the absorption of visible light from biospecimens, retaining the exploitation of the 532 nm laser for biological studies;^13^ (iv) the long time for images to be acquired due to the low Raman scattering cross section;^14^ (v) a noisy fluorescent background associated with the insulation material of the MEA, such as the silicon nitride, that hampers an integrated electrical and Raman imaging; (vi) further challenges corresponding to the customization of either instrument depending upon the available instrumentation. However, as we will show in this work, the road for the combination of MEA and Raman spectroscopy is indeed accessible.

Herein, we successfully combine electrophysiological activity monitoring using MEA and Raman measurements on neuronal rat primary cells, thus providing the first “proof-cased” integrated platform.

We demonstrated a tight correlation between neuronal firing rates and the intracellular levels of reduced cytochrome C that are monitored at the single-cell level by Raman spectroscopy. This finding establishes a foundation for a label-free and electrode-free optical method to assess neuronal firing activity.

To this aim, we have implemented a new protocol based on the repeated synchronous and sequential acquisitions of electrophysiological data and Raman spectra to explore any crosstalk of the two readouts.

Despite the exploitation of an energetic laser, such as the 532 nm wavelength excitation, the protocol successfully limits the laser-induced cell phototoxicity while maximizing signal quality and consistency between the data sets.

The peculiar crosstalk between the neuronal activity and the reduced cytochrome C content was further validated by comparison with different biospecimens, such as lipids, proteins, and nucleic acids. Notably, the dynamics of lipids, proteins, and nucleic acids upon chemical treatment of neuronal cultures can be further features to correlate to the drug treatment, highlighting the additional information provided with the integration of an optical and electrophysiological readout.

## Experimental Section

### Cell Culture and Media Compositions

Primary rat hippocampal neurons were purchased from Lonza (R-HI-501, Lonza Walkersville, United States). The recommended medium for this cell line is the PNGM Primary Neuron Growth Medium BulletKit (Lonza, Lonza Walkersville, United States). Since for Raman measurements, a medium without phenol red was needed, the neurobasal A medium without phenol red by Thermo Fisher Scientific was chosen. Neurobasal medium A without phenol red was supplemented with the B27 Supplement minus antioxidants (Thermo Fisher Scientific, Inc., Waltham, MA), l-glutamine (2 mM), gentamicin (50 μg/mL), amphotericin (37 ng/mL), and neural serum factor 1 (NSF1, 2%) (from the Lonza PNGM SingleQuots Growth Supplements).

Briefly, the chips were sterilized under UV exposure for 30 min under a cell culture hood. After sterilization, the culture surface area of the MEAs was coated with a solution of poly-d-lysine (30 μg/mL, Sigma-Aldrich, St. Louis, MO, USA) and laminin (2 μg/mL, Sigma-Aldrich, St. Louis, MO, USA) in phosphate-buffered saline (PBS, pH = 7.4, Thermo Fisher Scientific, Inc., Waltham, MA) for 1 h at room temperature to enhance cell adhesion and proliferation on the chips. Next, the devices were rinsed three times with sterile water (Sigma-Aldrich, St. Louis, MO, USA) and dried inside the hood before cell seeding. Primary rat hippocampal neurons were seeded on the devices and incubated (37 °C, 5% CO_2_, 95% humidity) for 4 h. Following adhesion, a portion of the medium was gently removed, and a fresh and prewarmed medium was introduced. Cell culture was sustained for 3 weeks. On day 5, 50% of the medium was replaced with a fresh and prewarmed medium, and the same medium change was performed every 3–4 days.

### Raman Spectroscopy

A Renishaw inVia instrument was used for Raman imaging. The samples were imaged using a 532 nm wavelength of excitation, a laser power of 13 mW at the objective, a 60× water immersive lens objective (NA = 1), a peak center set at 1200 cm^–1^, an integration time of 0.4 s, 1 accumulation, a step size acquisition of 1.5 or 3 μm, and high confocality mode. The size of the Raman maps collected in the sequential electrophysiological–spectroscopic analysis was 30 × 30 μm^2^. The required time for recording a 30 × 30 μm^2^ Raman map was 2.5 min.

### Data Processing

Raman data were collected and processed in two stages, and the preprocessing occurred using WiRE 5.5 and proceeded as follows: truncation, cosmic ray removal, noise filtering, baseline subtraction, and smoothing. The truncation was carried out maintaining the Raman spectrum from 500 to 1720 cm^–1^. The cosmic ray removal was carried out in two steps: (i) using a detection method with the width parameter and height parameter set as 3 and 15 and (ii) using the nearest neighbor algorithm with the noise level set as 0.89 and a scaling factor of 10 as well as the spectrum height set as 6.63 and a scaling factor of 50%. The noise filtering was based upon a WiRE 5.5 algorithm of principal component analysis, which results in the generation of a software interface in which the components (expressed as loadings) can be manually selected to discriminate the noise from the Raman signals. The subtraction of the baseline occurred using a polynomial order and noise tolerance of 12 and 1.5, respectively. Finally, the Raman spectra were smoothed using the Savitzky–Golay filter with a smooth window and polynomial order set as 9 and 3, respectively.

Then, the processed data were imported in MATLAB where they were further processed. The MATLAB script used was written implementing an existing one.^15^ Briefly, for the high-resolution Raman map illustrated in Figure 3, the Raman map was achieved by removing the noise associated with the substrate and media. This was achieved using a threshold approach that counts the number of pixels per point, and then, false-color images were generated normalizing the Raman intensity of interest to the peak of phenylalanine (about 1004 cm^–1^). For the Raman analysis, the interquartile range for each data set was calculated and the binning of the Raman spectra out of this range was performed. The preprocessed data set was then used to calculate the median corresponding to each Raman map. The accuracy in the calculation of the median Raman spectrum for each Raman map was therefore depending upon the binning threshold level and the interquartile range distribution. Then, the medians associated with a longitudinal recording were averaged to generate a representative Raman spectrum of the sample over time.

### Biocompatible Conditions

Special attention was given to maintaining the physiological conditions of the sample during the measurement. While the temperature (settled at 37 °C) was managed directly by the Multi Channel System (MCS GmbH), other parameters such as CO_2_ and humidity required the development of a custom chamber to be integrated between the Raman microscope and the Multi Channel System (MCS GmbH). This specially designed and conceived chamber allows the entrance of the observation objective from the top, and from the side wall, the flow of a heated and humidified 5% CO_2_–air mixture (provided by an Okolab system; Okolab-Stage Top-Digital Gas (oko-lab.com). The small losses of the chamber were compensated by the low incoming gas flow, so that a suitable condition for a long-term recording was reliably established. More details are available in the Supporting Information.

### Fluorescence Microscopy

The live–dead assay (L3224, Invitrogen) was carried out following the instructions from the manufacturer. Briefly, at the end of each experiment, neuronal rat cells were incubated with calcein AM (final concentration of about 2 μM) and ethidium homodimer-1 (final concentration of about 2.5 μM) for 30 min at room temperature and imaged without any washing attempts. Fluorescence microscopy was carried out using an EVOS Floid imaging system. Neuronal rat cells were focused using a 20× objective, while the measurements exploited fixed green and red LED light sources integrated with opportune readout filters. The collected data were imported into ImageJ to generate false-color images. The scale bar intensity was set from 0 to 4095 A.U., and the scale bar size was set to 125 μm.

### Electrophysiological Measurements

Electrophysiological measurements were carried out using a MEA2100 (MEA2100-LITE-System) utilizing TiN MEA with an electrode diameter of 30 μm and a spacing of 200 μm. High-pass and low-pass filters were set at 100 Hz (2^nd^ order) and 3500 Hz (4^th^ order), while the sample rate was set at 25 kHz. Data recordings were subsequently processed using customized Python code, enabling the calculation of metrics such as the firing frequency, average extracellular spike waveform, and spike count at a previously chosen electrode of interest. At the chosen electrode of interest, the detection of the single spikes was carried out using a threshold value exceeding 3 times the standard deviation of the background noise level. Thus, the firing rate accuracy in the section “The Definition of a Protocol for the Repeated Acquisitions of Electrophysiological Data and Raman Spectra” depends upon the chosen threshold level. The neuronal activity considering the whole active electrodes was calculated through the multichannel analyzer and using the default spike detection and analysis options. Specifically, the detection of the single spikes was based upon the negative phase of the neuronal spike and was carried out using a threshold value of 5 times the standard deviation of the background noise level. Thus, the firing rate accuracy in the section “Additional Information and Validation through the Integration of Electrophysiology and Raman Spectroscopy” depends upon this default threshold level. The electrophysiological recording was carried out for 60 s, and the calculated firing rates reflect the number of spikes per minute.

### Solutions Adopted to Integrate Electrical and Optical Measurements

The development of a transparent customized medium based upon the use of the neurobasal A medium supplemented with B27 minus antioxidants, l-glutamine (2 mM), gentamicin (50 μg/mL), amphotericin (37 ng/mL), and NSF1 (2%) enabled us to overcome the fluorescence noise associated with the designed commercially available PNGM Primary Neuron Growth Medium BulletKit from Lonza.

The overall weakness in Raman scattering was overcome focusing on the cytochrome C, a chromophore that is electronically excited using the 532 nm laser. Notably, the 532 nm laser excitation matches the energy difference between the excited and relaxed states of cytochrome C in the electronic Q-band transition.

The fluorescence background associated with the Raman measurements in the focal plane of the electrodes was circumvented carrying out focused Raman imaging. This also enabled us to minimize local heating and the cross interference between electrical and optical measurements as well as to preserve the electrodes from degradation.

Raman thermostable measurements were carried out using out-of-focus imaging and a chamber for “live-cell biocompatible” imaging conditions (37 °C and 5% CO_2_). This minimized the Raman spectra fluctuations dependent on temperature changes at the focal point.

The phototoxicity due to visible-light absorption of biospecimens was avoided thanks to prior calculations on the information redundancy originating from the Raman mappings. The definition of (i) the minimum Raman spectra to acquire and area to map as well as the largest step size to use without loss of significant information enabled us to minimize light absorption and local heating. This is clearly reflected by the presence of neuronal electrical activity at the end of each longitudinal measurement. Notably, neuronal activity is a well-known signature of living rat primary neuronal cells.

The potential crosstalk of electrical and optical measurements was overcome by exploiting a sequential approach in which electrical recording and Raman imaging were carried out in different times rather than simultaneously.

## Results and Discussion

### The Definition of a Protocol for the Repeated Acquisitions of Electrophysiological Data and Raman Spectra

Raman scattering is a rare phenomenon and results inversely related to the fourth power of the wavelength of excitation,^11,12^ hence the challenging exploitation of NIR lasers, such as 785 and 1064 nm, for any biological Raman readout. Due to these drawbacks and the increased Raman scattering of cytochrome C associated with its electronic Q-band transition,^16^ we exploited a 532 nm laser for the investigation of real-time, live-cell dynamics by Raman spectroscopy. Vibration modes that are coupled to the electronic transition are greatly enhanced in resonant Raman spectroscopy; in our case, it offered a richer readout on the excited-state structure of cytochrome C. The Raman scattering cross section in resonant Raman spectroscopy is commonly higher than several orders of magnitude.^17^ Notably, the development of a novel protocol enabled us to successfully limit the laser-induced cell phototoxicity while maximizing signal quality and consistency between the data sets (more details in the next section).

**Figure 1:**
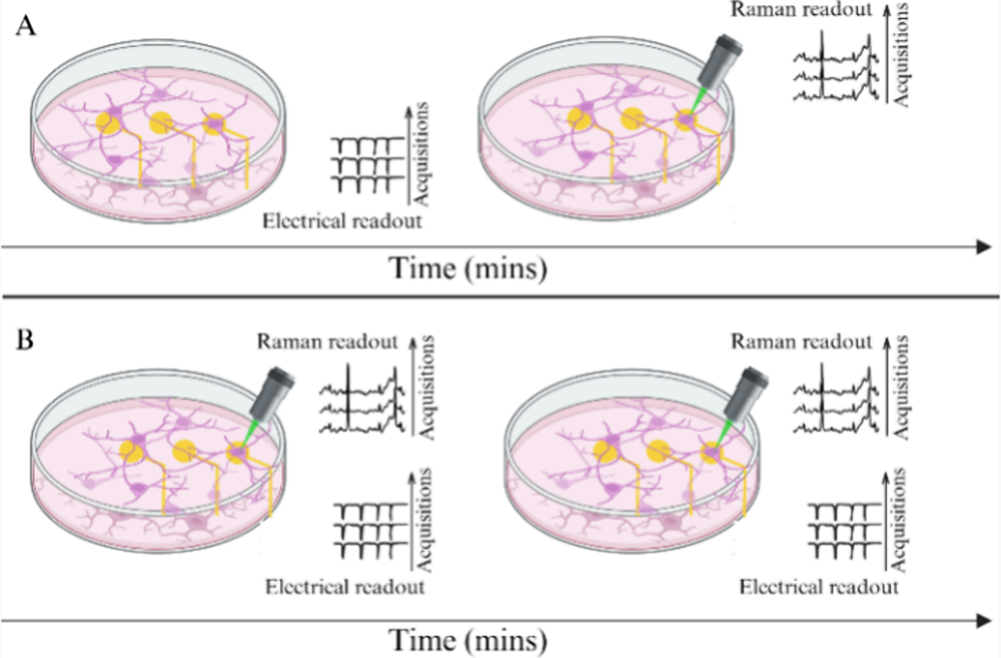
Development of a protocol for repeated (A) sequential or (B) synchronous acquisition of electrophysiological and spectroscopic data.

To accurately read out electrophysiological and spectroscopic features from rat hippocampal neurons, we have first developed a protocol to repeatedly monitor electrophysiological and spectroscopic features (Figure 1). With this aim, we acquired Raman spectra both synchronous and sequential with respect to electrical acquisitions.

**Figure 2:**
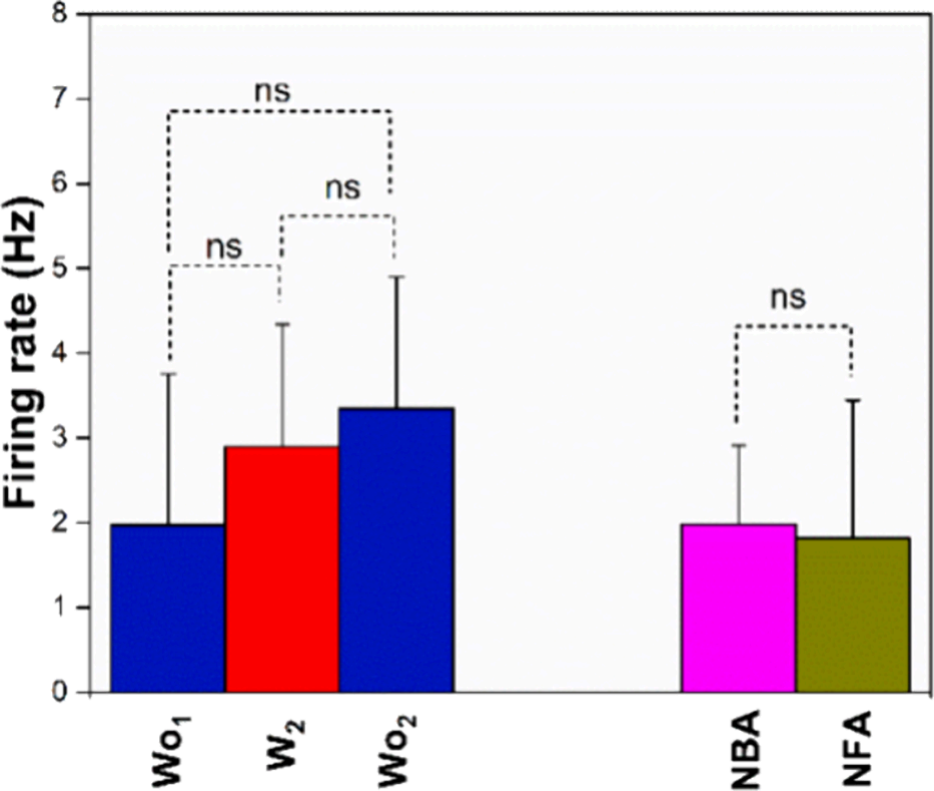
Effect of the laser illumination toward the neuronal firing rate. The neuronal firing rate before (Wo_1_), during (W_2_), and following (Wo_2_) the laser exposure (532 nm). The neuronal firing rate was measured at the beginning of the time lapse and then prior to any laser exposure (NBA) and the neuronal firing rate following 10 laser exposures (NFA).

As shown in Figure 1, the protocol used in this work adopts two monitoring case scenarios: (Figure 1A) the electrophysiological recording for 1 min prior to or following the acquisition of Raman data and (Figure 1B) the electrophysiological recording for 1 min during the acquisition of Raman data. Notably, the protocol exploits these two different approaches as the effect of the 532 nm laser illumination in the neighboring area of an active electrode and therefore over the neuronal electrical activity has to be characterized. Accordingly, we carried out repeated electrophysiological acquisitions in a sequential (Figure 1A) or synchronous (Figure 1B) fashion to unveil the effect of the 532 nm laser toward the electrical activity of a neuronal cell culture measured at a prior chosen active electrode. The platform used for such measurements is illustrated in Figure S1. The measured absolute values of the neuronal electrical activities over time, in the synchronous or sequential approach, are reported in Table S1. To assess the effect of the 532 nm laser toward the neuronal activity at the selected electrodes, we carried out a Mann–Whitney test between the neuronal activities values prior to or following laser illumination (Wo) and their values during laser exposure (*W*) (Figure 2). On the other hand, to exclude any potential phototoxicity induced by laser exposure, we carried out a Mann–Whitney test between the initial and final neuronal activities at the selected electrodes of reference, NBAs and NFAs, respectively (Figure 2).

The singular *p* values are reported in Table S2 and indicate the overall nonstatistically significant differences of the neuronal activity at the selected electrodes during laser illumination, prior to or following laser illumination and between the NBA and the NFA values of the neuronal cell cultures, indicating that the electrophysiological and spectroscopic readouts do not crosstalk and that the conditions used for the Raman acquisition do not induce any phototoxicity. These observations supported our choices on the use of commercially available transparent MEA as being responsible for negligible light absorption and local heating formation. The local heating was also circumvented by laser illumination in proximity close to the electrodes, not on the electrode itself, which is known to absorb visible light and produce heating,^18^ and further prevented by the out-of-focus imaging carried out during the Raman measurements, about +10 μm over the focal plane of the electrodes and the silicon nitride substrate, meaning of a lower energy density of the laser at the focal point of the electrodes. A lower energy density also preserved the electrodes from degradation due to high laser exposure.^18^ On that perspective, Altangerel *et al*. reported the use of a thin gold layer during a continuous laser exposure for facilitating the dissipation of thermal energy.^19^ However, this approach is not applicable to our MEA devices as the application of a gold layer between the electrodes would prevent the spatial readout of the neuronal electrical signaling due to short circuit between electrodes. Overall, the developed protocol does not affect the neuronal electrical activity that we aim to monitor, supporting the possible integration of Raman microscopy and electrophysiology for the investigation of functional and structural behavior in neuronal rat cells. Nonetheless, to exclude any possible interaction or artifacts among the neuronal activity and the laser, we opted for the most careful approach: the sequential recording of the neuronal electrical and vibrational activity. Accordingly, the following electrical activity and spectroscopic acquisitions were recorded in a sequential fashion, unless otherwise specified.

#### The Assignment of Vibrational Raman Bands in Neuronal Rat Cells

**Figure 3:**
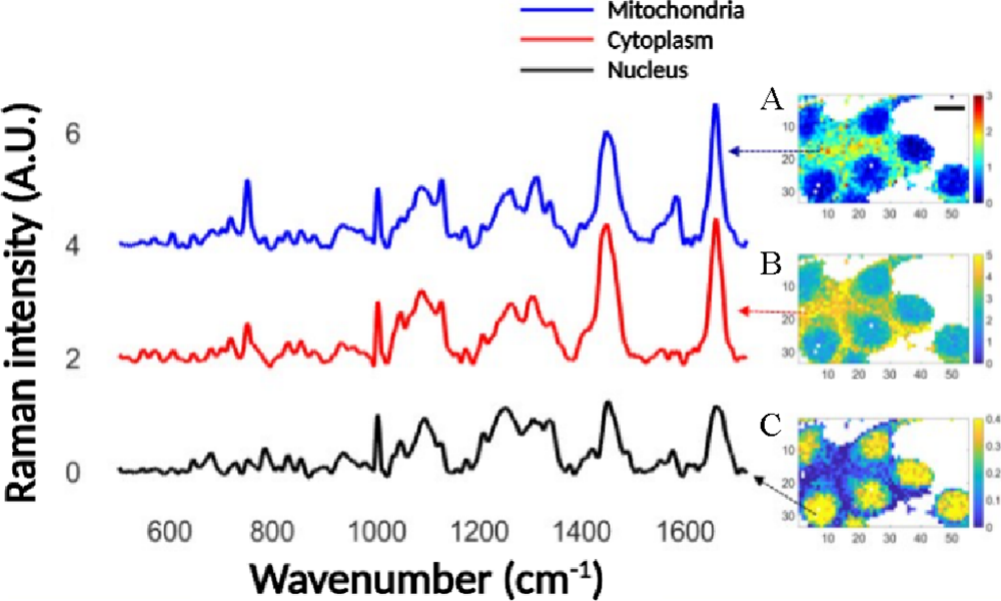
Raman fingerprints of neuronal rat cells in relation to the subcellular environments. (A) Raman map indicative of mitochondria and associated with the Raman ratio 750/1004 cm^–1^, (B) Raman map indicative of the cytoplasm and associated with the Raman ratio 1450/1004 cm^–1^, and (C) Raman map indicative of the nucleus and associated with the Raman ratio 782/1004 cm^–1^.

Electrogenic primary neuronal rat cells were cultured and measured on days *in vitro* (DIV) 21–25. As Raman scattering collection is hindered in colored fluorescent media,^20^ such as the phenol red-enriched medium provided by the cell manufacturer (Lonza), a customized medium for the R-HI-501 (Lonza) was designed using supplemented neurobasal medium A (transparent medium) suitable for long-term postnatal and adult brain neurons (more details in the methods and materials section). Raman scattering and fluorescence originate in a different timescale, which reflects the differences in the energy of the transitions.^21^ Indeed, methods such as time-gated Raman spectroscopy can discriminate the two signals; however, it is quite expensive with respect to conventional Raman spectroscopy. The latter, especially when using an excitation wavelength in the visible range, such as 532 or 633 nm, provides tangled spectra in which fluorescence often overwhelms the Raman signal.^21^ This phenomenon was also observed in our experiments, where live-cell Raman imaging was initially carried out in a phenol red-enriched medium (Lonza). For these reasons, we opted to use the custom transparent cell medium described above. Raman fingerprints of neuronal rat cells were acquired using a 532 nm wavelength of excitation, a laser power of 13 mW at the objective, a 60× water immersive lens objective with NA = 1, a peak center set at 1200 cm^–1^, an integration time of 0.4 s, 1 accumulation, a step size acquisition of 1.5 μm, and high confocality mode (Figure 3).

Figure 3 shows the normalized Raman fingerprints of neuronal rat cells. Notably, Raman spectra were normalized to the phenylalanine (about 1004 cm^–1^), a ubiquitous amino acid commonly used for relative quantification.^22^ The relative quantification to phenylalanine was chosen for two key reasons: (i) the signal of the amide I (about 1660 cm^–1^), commonly associated with proteins, which was observed as the strongest Raman signal, included a minimal contribution originating from the poly-d-lysine-laminin coating at the focal point of the electrodes, which was negligible at about 1004 cm^–1^; (ii) the Raman scattering of lipids, specifically the CH_2_ stretching (about 1450 cm^–1^), was found sensitive to changes under chemical pressure or physiological processes^23^ and therefore also excluded for relative normalization. In Raman microscopy, vector normalization is fundamental to ignore changes in Raman spectra due to different cell focusing and is overall common practice prior to any data analysis. Figure 3 shows that label-free ratiometric Raman imaging enables the distinction of vibrational features associated with the nucleus, cytoplasm, and mitochondria. Namely, the Raman scattering of nitrogenous bases occurs at 782 cm^–1^, the Raman scattering mostly related to lipids associated with −CH_2_ stretching occurring at 1450 cm^–1^, and the pyrrole breathing mode (ν_15_) in cytochrome C occurring at 750 cm^–1^. The Raman band at about 750 cm^–1^ is an established spectral signature associated with cytochrome C and mitochondria in nonapoptotic cells as previously exemplified by Yamakoshi *et al*.^24^ Notably, a weak Raman scattering occurring at 750 cm^–1^ can be also observed in the intranuclear region, and it is more likely related to the presence of cytochrome C above or below the nuclear focal plane according to the observations reported by Okada *et al*.^25^ Once the Raman signature of cytochrome C was established, resulting in a strong scattering at 750, 1127, 1316, and 1583 cm^–1^, we moved forward to minimize laser and area exposure for time lapse studies. The redundancy in Raman information across the cells was investigated by moving from high-resolution Raman maps to artificially created low-resolution Raman maps, considering the same scanning area. Notably, negligible differences were observed moving from a Raman map having 1.5 μm as the step size to 3 and 4.5 μm, suggesting redundancy of information (data produced using HeLa cells, not included). Hence, we opted to use 3 μm as the step size of acquisition and a minimum area of 30 × 30 μm^2^. Accordingly, the laser power and acquisition time for each spectrum were minimized down to 13 mW at the objective and 0.4 s, respectively. These precautions must be taken into account in Raman measurements as Raman spectra are temperature-dependent, and therefore, minimizing the spatiotemporal neuronal exposure is the key to a reliable measurement.^19^ The assignment of the Raman bands in neuronal rat cells enabled us to move forward in the integration of the electrical and spectroscopic readout, which as we mentioned above would adopt an out-of-focus Raman imaging to minimize local heating formation and preserve the integrity of the electrodes. Out-of-focus imaging and collection is key for two further reasons: (i) in excluding Raman changes triggered by the laser exposure itself due to the high-energy density of the laser at the focus spot and (ii) to exclude any optical background from the material of the MEA device (electrodes, tracks, and insulator). Our platform further minimized the Raman changes corresponding to the fluctuation of temperature, carrying out thermostable Raman measurements. Indeed, Raman imaging was carried out using a customized cell chamber for live-cell imaging (37 °C, 5% CO_2_).

#### Additional Information and Validation through the Integration of Electrophysiology and Raman Spectroscopy

**Figure 4:**
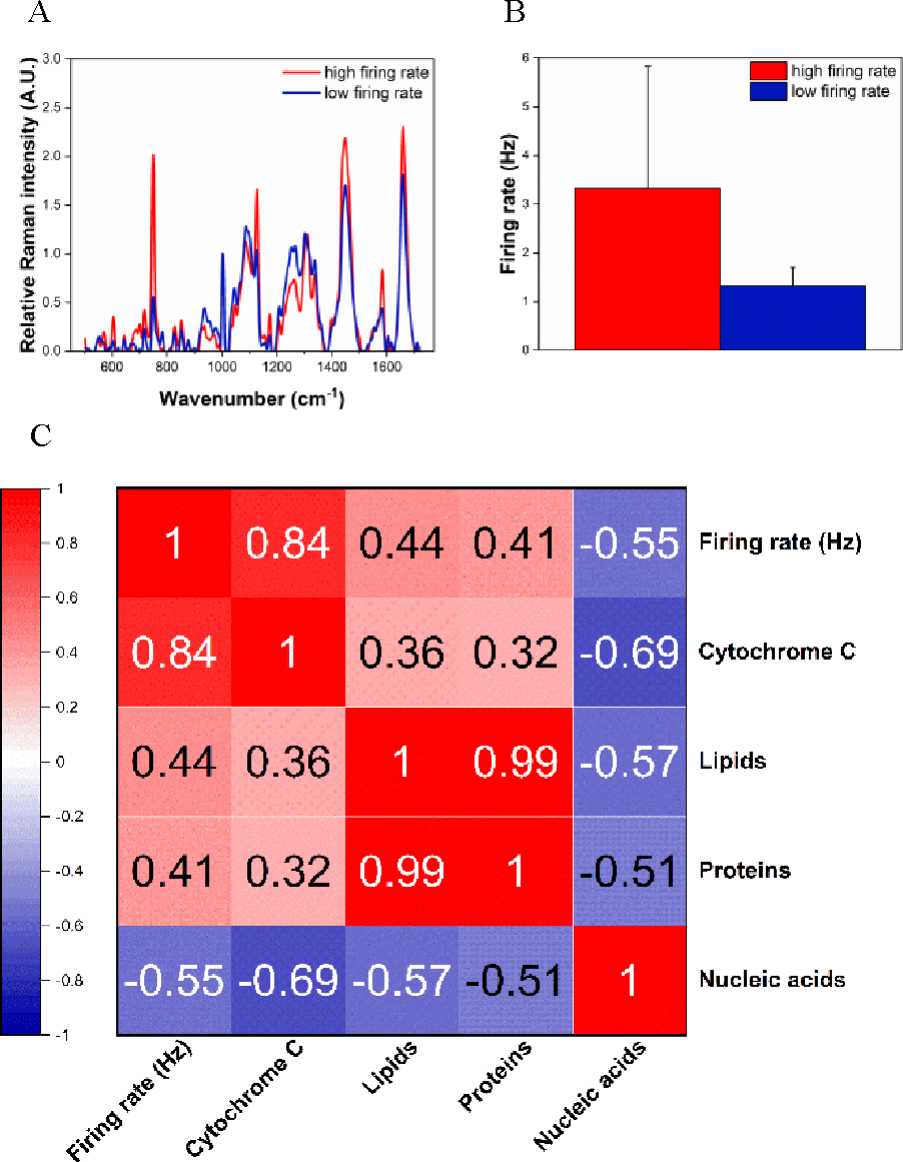
Molecular changes associated with the neuronal firing rate. (A) Representative longitudinal average Raman spectra associated with a high and a low neuronal firing rate plotted against (B) their respective neuronal firing rate; we illustrate the representative sample for the high and low neuronal firing rate in red and blue, respectively. Data processing and analysis were carried out according to Figures S2 and S3. (C) Correlation matrix between the neuronal firing rate, the reduced cytochrome C content (750 cm^–1^), proteins (1660 cm^–1^), lipids (1450 cm^–1^) and nucleic acid content (782 cm^–1^). In hot colors and blue colors, we expressed the high- and low-correlation Pearson values, respectively. The correlation is calculated over seven different measurements and more than three different neuronal cell cultures.

Electrical and optical label-free integration may open the gate for large improvements in functional and structural toxicity and overall in the much more in-depth characterization of biological models. While the electrical activity of the rat neuronal cells reflects cell communication at the cellular and network level, the phenotypical Raman readout in proximity of the electrode of interest reaches a subcellular resolution, enabling the monitoring of intracellular dynamics such as metabolic pathways, oxidative stress, apoptosis, and more. As a proof of concept, neuronal rat cells were cultured on a commercially available TiN MEA, and sequential electrical–spectroscopical measurements were carried out accordingly to the protocol reported in Figure 1B. Specifically, we collected “out-of-focus” Raman spectra in the form of 30 × 30 μm^2^ Raman maps in between electrophysiological acquisitions. Notably, the electrophysiological data analysis was carried out considering all of the active electrodes of the MEA device. The Raman maps were collected using a 532 nm wavelength excitation laser, a power of 13 mW at the objective, an immersive lens objective 60× Nikon with NA = 1, an integration time of 0.4 s, a peak center set to 1200 cm^–1^, a step size of 3 μm, and high confocality conditions. The Raman spectra associated with each map were processed using WiRE 5.5, imported in MATLAB and normalized to the internal peak corresponding to the phenylalanine (1004 cm^–1^). The interquartile range for each data set was calculated, and the binning on the Raman spectra out of this range was performed. The preprocessed data set was then used to calculate the median corresponding to each Raman map. The firing rates and the median Raman spectra of each time lapse were extracted and averaged as reported in Figures S2 and S3, respectively. The extent of molecular changes associated with a different neuronal firing rate is exemplified in Figure 4 where two representative Raman spectra (Figure 4A) are plotted against their respective neuronal firing rate (Figure 4B).

Once the average firing rate and Raman spectrum for each time lapse were calculated, we correlated the biological features available using our integrated platform, namely, the neuronal firing rate (Hz) and the reduced cytochrome C (750 cm^–1^), proteins (1660 cm^–1^), lipids (1450 cm^–1^), and nucleic acid content (782 cm^–1^). The correlation matrix of these biological features is shown in Figure 4C.

Figure 4A,B illustrates the extent of molecular changes associated with the high and low neuronal firing rate in neuronal rat cells. Specifically, the high firing rate was accompanied by an overall higher content of the biospecimen, suggesting that a higher metabolic activity at the single-cell level may reflect the electrophysiological activity across the whole cell culture. To further unveil any correlation patterns among the biological variables, we carried out a correlation analysis within the whole data set. Figure 4C shows a positively strong correlation between the neuronal firing rates and the reduced cytochrome C contents (Pearson value = 0.84, *p* value = 0.019). This correlation can be reasoned by the high dependence of the neuronal activity from the ATP synthesis produced by the OxPhos. Therefore, this suggests the use of resonant Raman microscopy and the longitudinal monitoring of the reduced cytochrome C as alternative tools for an electrode-free and label-free assessment of the electrophysiological firing rate and the overall health status of neuronal cell cultures in the presence of electrical noise and high electrical impedance as well as for the label-free monitoring of the electrophysiological firing rate at the single-cell level prior to and following drug exposure. We speculate that a high neuronal activity decreases the intracellular (ATP/ADP) ratio, which drives the positive modulation of the ETC and, more specifically, the cytochrome C oxidase. In support of this hypothesis, cytochrome C oxidase is a well-established biomarker of neuronal health.^26^

Conversely to the reduced cytochrome C content, lipids, proteins, and nucleic acids do not show any correlation with the neuronal firing rate. While proteins and lipids strongly correlate between them (Pearson value = 0.99, *p* value = 6 × 10^–6^), the nucleic acids do not correlate with any monitored biological feature. The feature correlation lays the foundation for an alternative approach to the electrophysiological firing rate via Raman spectroscopy and the monitoring of the *denovo* synthesis of lipids, proteins, and nucleic acids for shedding light on drug-induced dynamics at the single-cell level. Clearly, the advantages in the integration of electrical and spectroscopic readout in assessing the structural and functional neurotoxicity are shown. Furthermore, the single-cell resolution of the spectroscopic measurement also enables one to assess the different drug responsiveness of single cells following different intracellular drug accumulations or cellular phenotypes.

To further confirm the “biocompatibility” of our technique, a live/dead assay following the 10 sequential measurements was carried out in some of the samples (Figures S4–S6). Notably, the staining with calcein AM and ethidium homodimer-1 indicated that neither the time onto the stage nor the excitation at 532 nm triggered cell death, with the more likely dead cells in the samples corresponding to the impossibility of spinning down the cells prior to cell seeding and going accordingly with the unceasing neuronal activity observed at the end of each time lapse.

Overall, the implementation of electrophysiological techniques with an optical imaging analysis such as Raman spectroscopy paves the way for a more accurate neurotoxicity screening within the drug development pipeline with a potential translation in clinics using optical fibers^27^ and biocompatible implantable devices^28^ as well as in the investigation of neurodevelopmental disorders or neurodegenerative diseases. The integration of the two approaches likely enables one to follow neurotoxicity dynamics in either case: (a) changes in the electrophysiological activity in low electrical noise and impedance, (b) changes in the electrophysiological activity in high electrical noise and impedance monitoring the reduced cytochrome C content, and (c) the drug-induced dynamics over the *denovo* synthesis of proteins, lipids, and nucleic acids.

Further steps of this work will be exploiting this novel technology for (i) inducing changes in the electrical activity of the neuronal rat cells and comparing the accuracy in the assessment of the neurotoxicity using lone electrophysiological measurements, Raman measurements, and finally the integrated platform, (ii) inducing oxidative stress to the neuronal cell cultures to verify if the loss in feature correlation may be a valid tool for early induced neurotoxicity assessment, (iii) the monitoring of the electrical and Raman activity in long-term studies, and (iv) the use of Raman-tagged drugs to cross-correlate the several biological features with the overall drug uptake. We also envision the integration of coherent Raman spectroscopy and electrophysiological techniques, with clear advantages dependent on the use of longer wavelengths and the coherent in-phase oscillations of photons, resulting in increased sensitiveness of detection, less chance to induce phototoxic effects, and less light scattering over distance enabling an in-depth investigation across 3D biological samples.

## Conclusions

Herein, we describe a novel tool to longitudinally investigate electrophysiological and Raman activity within electrogenic cell monolayers. This approach paves the way for the novel capabilities of multiplexed and longitudinal investigations of neuronal cell culture and neurotoxicity. Notably, the method is label-free and nondestructive and in perspective can find applications in those fields in which the administration of dyes or labels is not recommended, such as in drug discovery and development. The proposed method enables a better understanding of biological dynamics in drug testing, relating drug cytotoxicity to the changes in the electrophysiological activity of the sample and the following of metabolic activity and biological specimens across the sample for in-depth characterization and developmental studies. We showcased the advantages of this novel technology demonstrating the tight correlation between the upstream and downstream features of the OxPhos, the reduced cytochrome C, and the neuronal firing rate. Thus, this proves that the electrophysiological firing rates can be monitored at the single-cell level via electrode- and label-free mean Raman spectroscopy. A key approach is in the presence of high electrical noise and impedance during the measurements. Furthermore, we have demonstrated the advantages of the integration of MEA and Raman measurements by the collection of the uncorrelated Raman scattering originating from lipids, proteins, and nucleic acids, which opens the possibility of monitoring the *de novo* synthesis of biospecimens as an additional perspective on neurotoxicity. Further advancements of this technology can be achieved by taking advantage of coherent Raman spectroscopy or time-gated spectroscopies, even though they require much higher costs.

## Supporting Information Available

Materials and methods, instrumentation used for this work, data processing, and analysis and experimental details (PDF)

## Abbreviations

MEA: multielectrode array
TiN: titanium nitride
DIV: days *in vitro*
PNGM: primary neuron growth medium
NSF1: neural serum factor 1
PBS: phosphate-buffered saline
ETC: electron transport chain
OxPhos: oxidative phosphorylation
