# Supplementary material for: Electrode- and Label-Free Assessment of Electrophysiological Firing Rates through Cytochrome C Monitoring via Raman Spectroscopy
Source: ACS Sens. 2025 Feb 5;10(2):1228–36. doi: 10.1021/acssensors.4c03133 (PMC11877498; doi:10.1021/acssensors.4c03133)
Supplement: Supplementary file 1 — se4c03133_si_001.pdf [file se4c03133_si_001.pdf]

Electrode- and label-free assessment of electrophysiological firing rates through cytochrome C monitoring via Raman spectroscopy.

Electronic Supplementary information

Christian Tentellino,[1*] Marta d'Amora,[1,2] Rustamzhon Melikov,[1] Giuseppina Iachetta,[1] Giulia Bruno,[1] Francesco Tantussi,[1] Michele Dipalo[1] and Francesco De Angelis[1*]

[1]Istituto Italiano di Tecnologia, Department of Plasmon Nanotechnologies, Via Morego 30, Genoa, 16153, Italy

[2]University of Pisa, Department of Biology, S.S. 12 Abetone e Brennero, 4 , Pisa, 56127, Italy

Contents

Material and Methods……………………………………………………………………………..2

Figure S1-S6……………………………………..……………………......……………..……………….5

Material and methods

Cell Culture and media compositions

Primary rat hippocampal neurons were purchased from Lonza (R-HI-501, Lonza Walkersville, United States). The recommended medium for this cell line is the PNGM™ Primary Neuron Growth Medium BulletKit™ (Lonza, Lonza Walkersville, United States). Since for Raman measurements, a medium without phenol red was needed, the Neurobasal Medium A without phenol red by Thermo Fisher Scientific was chosen. Neurobasal medium A was supplemented with the B27 minus antioxidants Supplement (Thermo Fisher Scientific, Inc., Waltham, MA), L-glutamine (2 mM), gentamicin (50 µg/mL), amphotericin (37 ng/mL) , and Neural Serum Factor 1 (NSF1, 2%)(from the Lonza PNGMTM Singlequots™ Growth Supplements).

Briefly, the chips were sterilized under UV exposure for 30 mins under a cell culture hood. After sterilization, the culture surface area of the MEAs was coated with a solution of poly-D-lysine (30 µg/mL ,Sigma-Aldrich, St. Louis, MO, USA) and laminin (2 µg/mL, Sigma-Aldrich, St. Louis, MO, USA) in Phosphate Buffer Saline (PBS, pH = 7.4, Thermo Fisher Scientific, Inc., Waltham, MA) for 1 h at room temperature, to enhance cells adhesion and proliferation on the chips. Next, the devices were rinsed three times with sterile water (Sigma-Aldrich, St. Louis, MO, USA) and dried inside the hood, before cell seeding. Primary rat hippocampal neurons were seeded on the devices and incubated (37 °C, 5% CO2, 95% humidity) for 4 h. Following adhesion, a portion of the medium was gently removed, and fresh and pre-warmed medium was introduced. Cell culture was sustained for 3 weeks. On day 5, 50% of the medium was replaced with fresh and pre-warmed medium and the same medium change was performed every 3-4 day.

Raman spectroscopy

A Renishaw InVia was used for Raman imaging. The samples were imaged using a 532 nm wavelength of excitation, laser power of 13 mW at the objective, a 60x water immersive lens objective (NA=1), peak centre set at 1200 cm^{-1}, integration time of 0.4 seconds, 1 accumulation, step size acquisition of 1.5 or 3 µm and high confocality mode. The size of the Raman maps collected in the sequential electrophysiological-spectroscopic analysis was 30x30 μm^2.

Data processing

Raman data were collected and processed in two stages, the preprocessing occurs using WiRE 5.5 and proceeds as follows: truncation, cosmic rays removal, noise filter, subtraction baseline and smooth. The truncation was carried out maintaining the Raman spectrum from 500 to 1720 cm^{-1}. The cosmic rays removal was carried out in two steps, (i) using a detection method with width parameter and height parameter set as 3 and 15 and (ii) using the nearest neighbour algorithm with noise level set as 0.89 and scaling factor 10 as well as spectrum height set as 6.63 and scaling factor 50%. The noise filter was based upon a WiRE 5.5 algorithm of principal component analysis which results in the generation of a software interface in which the components (expressed as loadings) can be manually selected to discriminate the noise from the Raman signals. The subtraction of the baseline occurred using polynomial order and noise tolerance as 12 and 1.5, respectively. Finally, the Raman spectra were smoothed using the Savitski-Golay filter with smooth window and polynomial order set as 9 and 3, respectively. Then, the processed data were imported in MATLAB where the data were further processed. The MATLAB script used was written implementing an existing one.[16] Briefly, for the high-resolution Raman map illustrated in Figure 3, the Raman map was achieved by removing the noise

associated with the substrate and media. This was achieved using a threshold approach which counts the number of pixels per point, and then false-color images were generated normalizing the Raman intensity of interest to the peak of phenylalanine (about 1004 cm^{-1}). For the Raman analysis, the interquartile range for each data set was calculated and the binning of the Raman spectra out of this range was performed. The pre-processed data set was then used to calculate the median corresponding to each Raman map. Then, the medians associated with a longitudinal recording were averaged to generate a representative Raman spectrum of the sample over time.

Biocompatible conditions

Special attention has been given to maintaining the physiological conditions of the sample during the measurement. While the temperature (settled at 37 °C) is managed directly by the Multi Channel System MCS GmbH, other parameters such as CO_2 and humidity, required the development of a custom chamber to be integrated between the Raman microscope and the Multi Channel System MCS GmbH. This specially designed and conceived chamber allows the entrance of the observation objective from the top, and from the side wall, the flow of heated and humidified 5% CO_2-Air mixture (provided by an Okolab system; Okolab - Stage Top - Digital Gas (oko-lab.com). The small losses of the chamber are compensated by the low incoming gas flow so that a suitable condition for a long-term recording is reliably established.

Fluorescence microscopy

The live-dead assay (L3224, Invitrogen) was carried out following the indications from the manufacturer. Briefly, at the end of each experiment neuronal rat cells were incubated with Calcein AM (final concentration about 2 µM) and Ethidium homodimer-1 (final concentration about 2.5 µM) for 30 minutes at room temperature and imaged without any washing attempts. The fluorescence microscopy was carried out using a EVOSTM Floid imaging system. Neuronal rat cells were focused using a 20x objective while the measurements exploited fixed green and red LED light sources integrated with opportune readout filters. The collected data were imported in ImageJ to generate false-color images. Scale bar intensity was set from 0 to 4095 A.U. and scale bar size was set to 125 µm.

Electrophysiological measurements

Electrophysiological measurements were carried out using a MEA2100 (MEA2100-LITE-System) utilizing TiN MEA with electrode diameter of 30 µm and spacing of 200 µm. High pass and low pass filters were set at 100 Hz (2nd order) and 3500 Hz (4th order) while the sample rate was set at 25 kHz. In the analysis of the interference between light illumination and electrical recordings, electrophysiological data were processed using customized Python software, enabling the calculation of metrics such as firing frequency, average extracellular spike and spike count considering the only electrode of interest. Conversely, the sequential approach in the section:" The additional information and validation through the integration of electrophysiology and Raman spectroscopy" considered all active electrodes. The neuronal activity considering the whole active electrodes was calculated through the Multi Channel Analyzer and using the default spike detection and analysis options. We supervised the spike detection and analysis by the removal of the signal associated with the electrical noise.

The integrated platform for electrophysiological and Raman readout

The schematics of the instrument used for the electrophysiological and Raman measurements are shown in Figure S1. Briefly, it consists of a “customized” Renishaw InVia Raman microscope implemented with a Multi Channel System MCS GmbH. While the Multi Channel System MCS GmbH allows the control of temperature (37°C), other incubator-like conditions such as 5% CO_2 and humidity were reproduced thanks to a customized chamber having both inlet and outlet channels. The inlet channel is represented by a heated tube coupled to a line gas controller through an active humidity controller (Okolab; (oko-lab.com)). The line gas controller settings are set to create a 5% CO_2 gas which is humified by flowing into warm water in the proximity of the active humidity controller. Then, the humified gas flows over a heated tube until reaching the sample chamber located on the stage of the InVia Renishaw Raman microscope. The enclosed chamber on the stage is built having the Multi Channel System MCS GmbH on the base, a hollow-concentric chamber hosting the TiN MEA and the inlet channel. The “ceiling” of the enclosed chamber is composed of two magnetic complementary elements (united during measurements) surrounding the water-immersive objective lens 60x (Nikon, NA=1) used to focus and light the sample with a monochromatic wavelength excitation and collect the back-scattered light originating from the sample. The shape of the two magnetic complementary elements is designed to have a larger diameter than the water-immersive objective lens to enable movements in the x-y axis during the acquisition of Raman maps and avoid condensation. Notably, the magnetic elements are silicon-based and easily sterilized using ethanol.

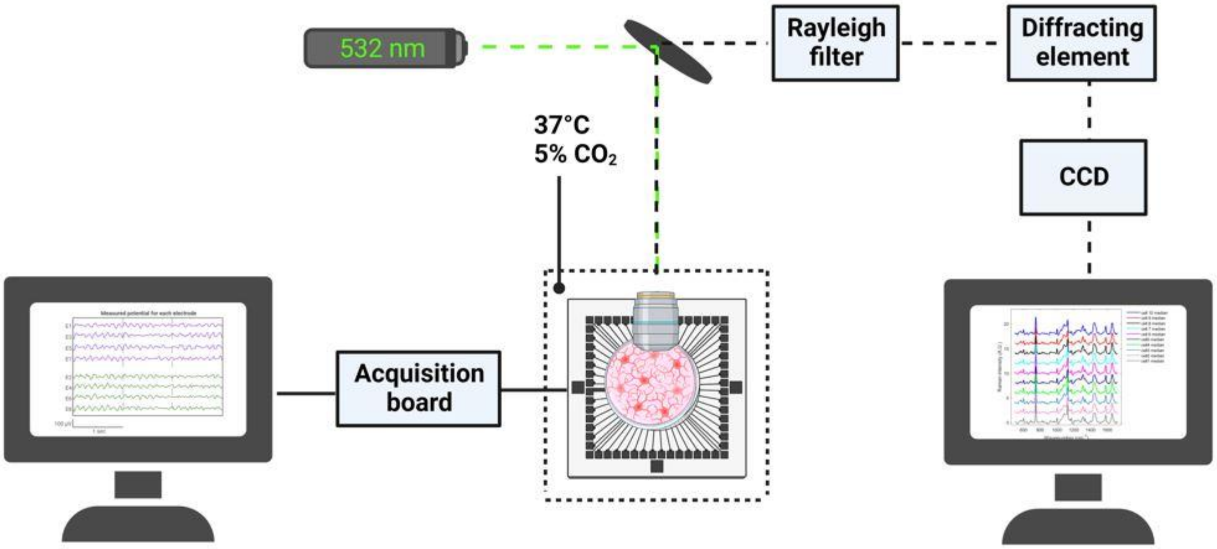

Figure S1. The schematics of the integration of a customized Renishaw InVia Raman microscope and a Multi Channel System MCS GmbH for the sequential electrophysiological and Raman measurements.

Time (mins)

	Sample							
	1	2	3	4	5	6	7	8
**NBA**	0.64	2.23	3.57	0.84	1.88	3.04	1.59	2.04
**W_1**	1.28	3.12	5.02	2.75	5.31	4.92	0.55	3.31
**Wo_1**	0.63	2.07	5.74	0.06	2.46	3.34	0.13	1.30
**W_2**	1.44	2.85	4.92	4.11	1.72	1.23	4.91	1.99
**Wo_2**	1.31	3.43	6.13	2.78	3.65	5.28	1.83	2.39
**W_3**	1.65	1.61	3.20	2.14	1.58	2.17	1.28	3.05
**Wo_3**	0.56	4.08	5.17	4.22	2.89	3.75	2.76	2.03
**W_4**	1.69	2.13	5.61	2.04	2.76	4.43	2.77	1.83
**Wo_4**	1.16	2.25	6.12	1.58	1.35	1.09	1.85	1.65
**W_5**	1.26	3.57	4.52	4.04	2.80	5.22	2.21	1.52
**Wo_5**	1.09	2.26	4.36	3.23	1.08	2.29	1.00	1.64
**W_6**	1.53	2.46	9.97	1.39	1.90	3.19	1.65	1.19
**Wo_6**	1.41	2.26	4.01	3.48	1.72	5.72	3.70	1.13
**W_7**	1.93	2.23	4.79	3.77	0.92	2.06	0.25	0.97
**Wo_7**	1.01	2.65	4.97	2.19	0.59	3.46	2.29	0.53
**W_8**	1.58	2.75	3.07	3.67	1.06	4.86	1.85	0.33
**Wo_8**	1.39	1.72	4.82	2.75	1.21	0.96	0.13	0.47
**W_9**	1.14	3.14	4.46	0.52	0.46	5.90	0.72	0.58
**Wo_9**	1.62	2.79	2.21	4.67	0.20	2.02	2.80	0.34
**W_{10}**	0.79	3.03	3.15	3.97	0.04	3.58	1.25	0.60
**NFA**	1.67	2.17	1.92	0.08	0.12	5.43	2.56	0.56

Table S1. Changes in the absolute value of spike frequency of the neuronal rat cell culture prior to or following laser exposure over time extracted from a previously selected active electrode. Eight different neuronal cell culture samples were measured. The neuronal activity at the beginning and at the end of the time-course experiment are reported as NBA and NFA, respectively. The neuronal electrical activity over time recorded in the presence or the absence of laser exposure are reported as W and Wo, respectively. The exposure to the laser reproduces Raman maps acquisition-like conditions such as 532nm laser (13 mW at the objective), acquisition time 0.4s, step size 3 μm, immersive objective lens (60x), 30 x 30 $μm^2$, high confocality.

**Mann-Whitney test**	**P value**
NBA vs W_1	0.1562 (^{ns})
W_1 vs Wo_1	0.2271 (^{ns})
Wo_1 vs W_2	0.3184 (^{ns})
W_2 vs Wo_2	0.5635 (^{ns})
Wo_2 vs W_3	0.0831 (^{ns})
W_3 vs Wo_3	0.1035 (^{ns})
Wo_3 vs W_4	0.5635 (^{ns})
W_4 vs Wo_4	0.0660 (^{ns})
Wo_4 vs W_5	0.1565 (^{ns})
W_5 vs Wo_5	0.1565 (^{ns})
Wo_5 vs W_6	0.6365 (^{ns})
W_6 vs Wo_6	0.4948 (^{ns})
Wo_6 vs W_7	0.3184 (^{ns})
W_7 vs Wo_7	0.7132 (^{ns})
Wo_7 vs W_8	0.7929 (^{ns})
W_8 vs Wo_8	0.2271 (^{ns})
Wo_8 vs W_9	1.0000 (^{ns})
W_9 vs Wo_9	1.0000 (^{ns})
Wo_9 vs W_{10}	0.8748 (^{ns})
W_{10} vs NFA	0.6365 (^{ns})
NFA vs NBA	0.5635 $^{(ns)}$

Table S2. P values of the Mann-Whitney tests when the neuronal electrical activity during the Raman imaging acquisition is compared to the neuronal electrical activity prior to or following the Raman imaging acquisition. The calculations were carried out using the absolute values of the neuronal activity measured at the active electrode of reference reported in table S2. Eight different samples were used for the Mann-Whitney tests calculations. Significance was set as equal to or lower the 0.05 level(*).

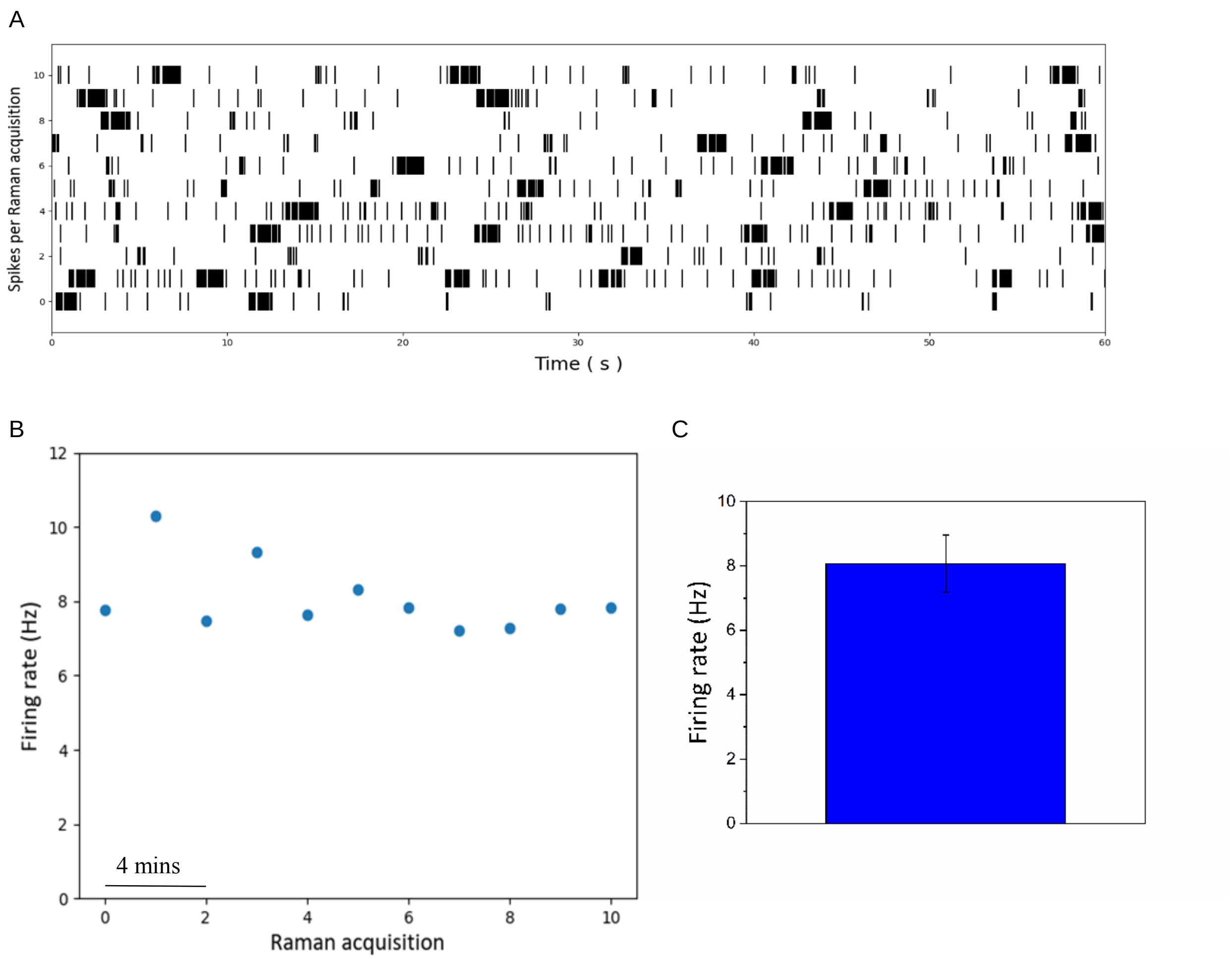

Figure S2. Data processing steps in the calculation of the averaged firing rates of the neuronal primary cell culture. (A) The first step of each electrical recording expects the measurements of the single neuronal spikes over time. (B) Then, the single neuronal spikes of each measurement are used to calculate the firing rate of the neuronal activity corresponding to each recording.(C) The all frequency rates corresponding to the longitudinal measurement are averaged to calculate a representative neuronal firing rate of the sample.

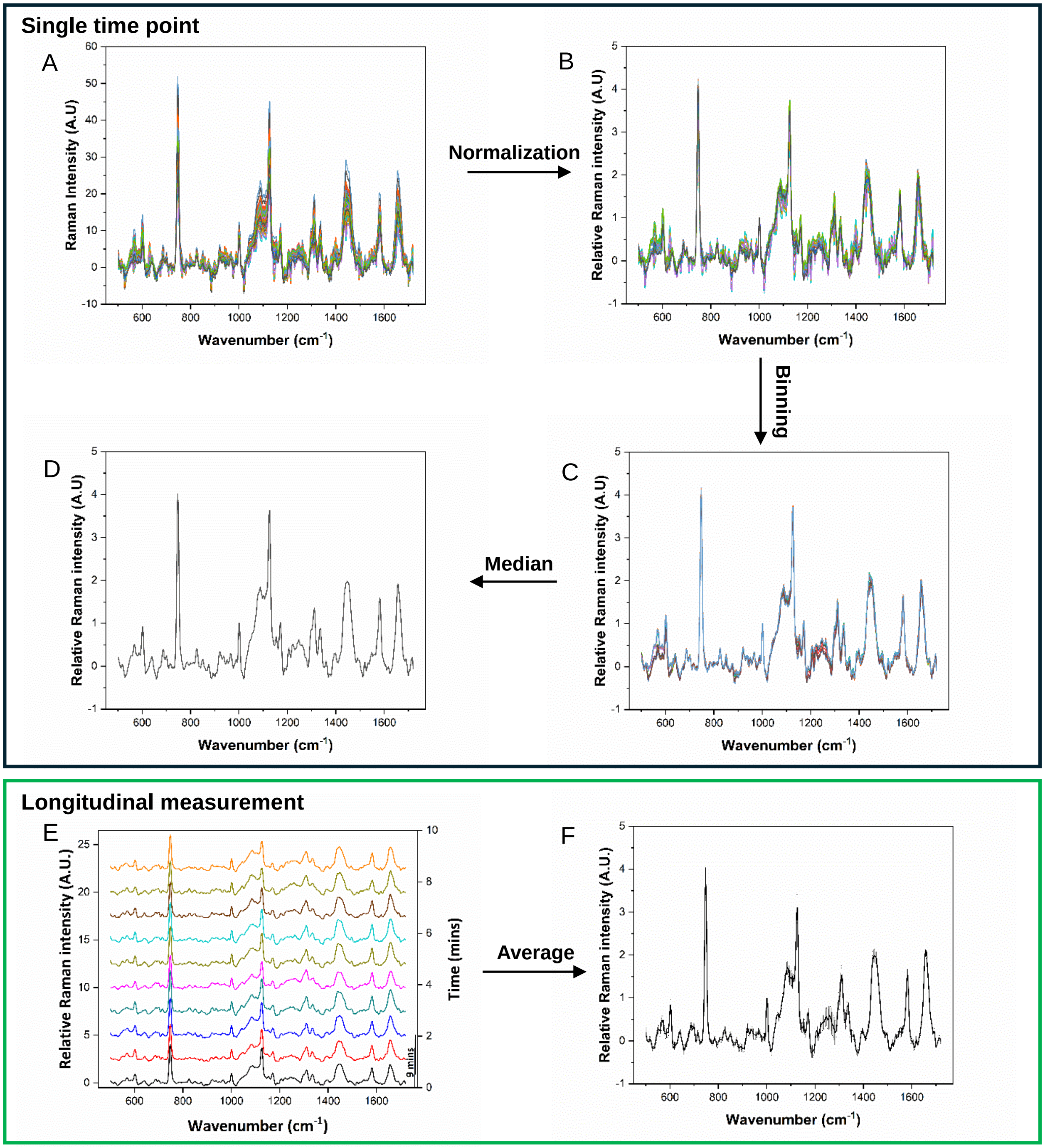

Figure S3. Raman medians of the same mapping area over time. Each map was collected in the proximity close area to the electrode of interest and between two electrical activity recordings. The Raman maps were collected using a 532nm wavelength excitation laser, power of 13 mW at the objective, immersive-lens objective 60x Nikon with NA=1, integration time of 0.4 s, peak center set to 1200 cm^{-1}, step size of 3 μm and high confocality conditions. (A) The Raman spectra associated with each map were extracted and (B) normalized to the internal peak corresponding to the phenylalanine (1004 cm^{-1}). (C) The interquartile range for each data set was calculated and the binning on the Raman spectra out of this range was performed. (D) The pre-processed data set was then used to calculate the median corresponding to each time point within the longitudinal recording. (E) The medians of the longitudinal recording are then averaged to achieve a representative average Raman spectrum of the sample over time (F).

A

B

C

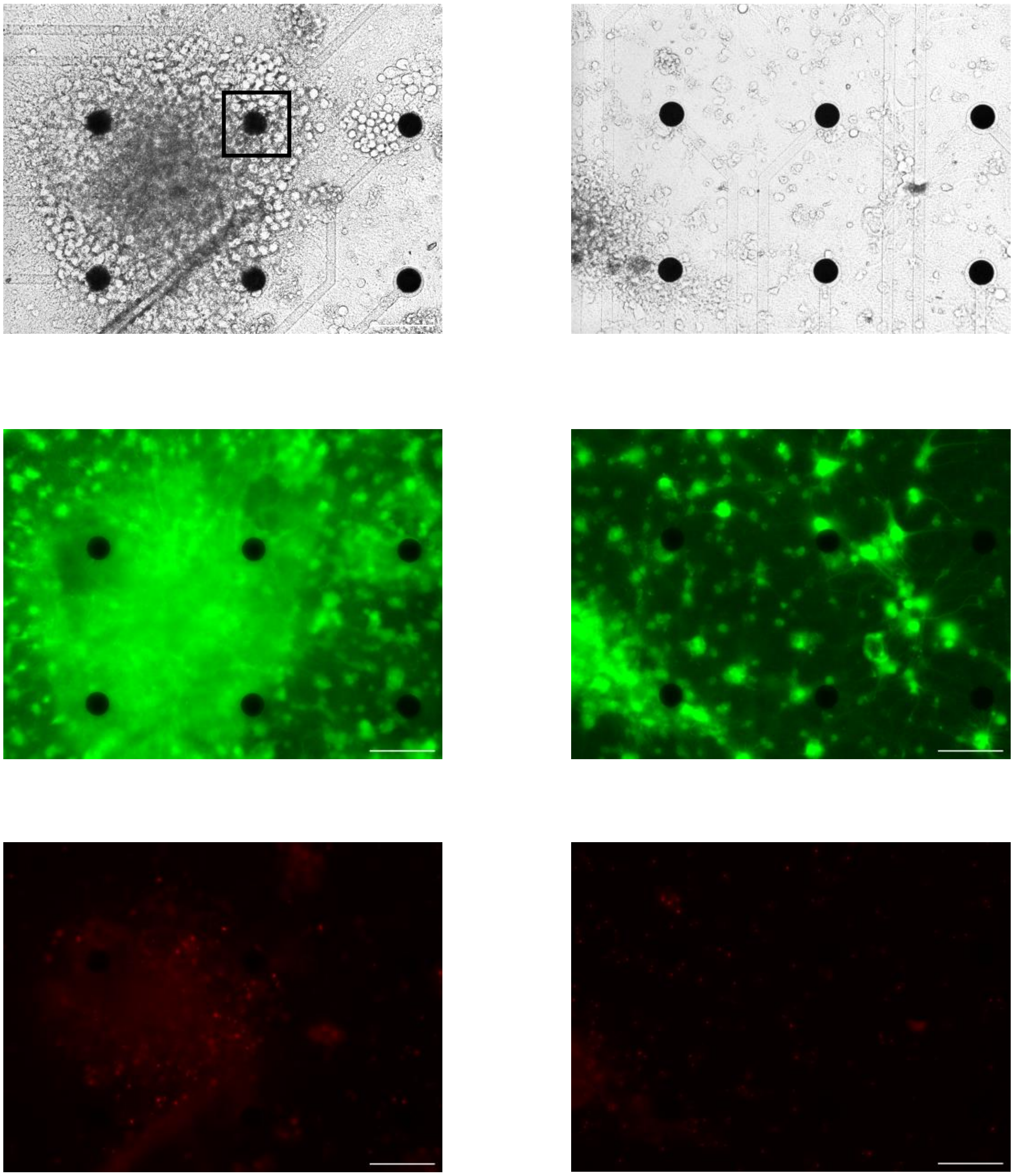

Figure S4. Live-dead assay of neuronal rat cells at the end of a longitudinal experiment. The concentration and incubation time for calcein and ethidium homodimer were chosen according to the recommendations of the manufacturer. (A) Bright-field image, enclosed in a square the area of interest that was used for running the integrated, sequential electrophysiological-spectroscopic analysis. (B) Calcein-image indicating live cells. (C) Ethidium-homodimer-image indicating dead cells. Scale bar size: 125 μm. Scale bar intensity: 0-4095 A.U.

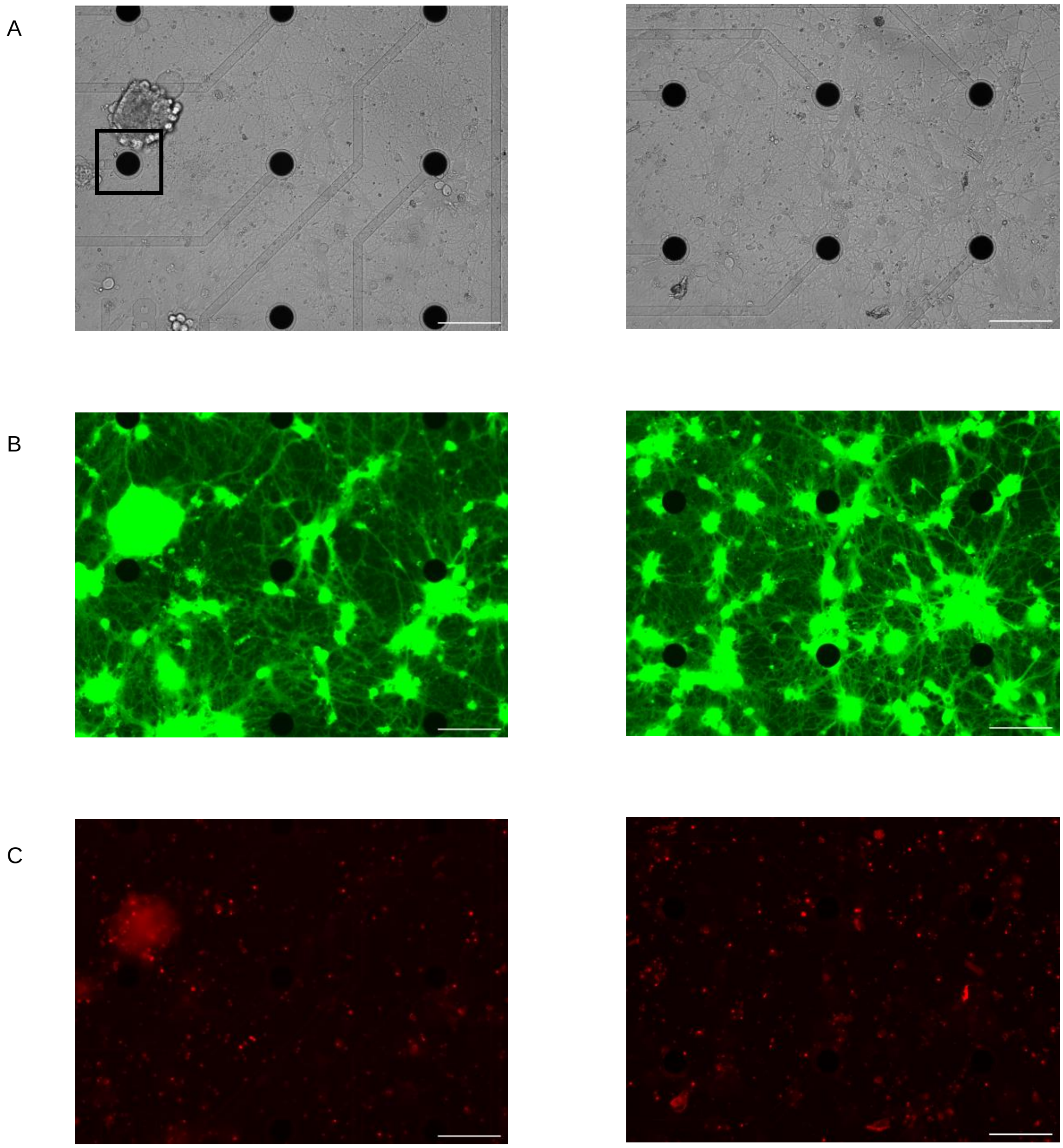

Figure S5. Live-dead assay of neuronal rat cells at the end of a longitudinal experiment. The concentration and incubation time for calcein and ethidium homodimer were chosen according to the recommendations of the manufacturer. (A) Bright-field image, enclosed in a square the area of interest which was used for running the integrated, sequential electrophysiological-spectroscopic analysis. (B) Calcein-image indicating live cells. (C) Ethidium-homodimer-image indicating dead cells. Scale bar size: 125 µm. Scale bar intensity: 0-4095 A.U.

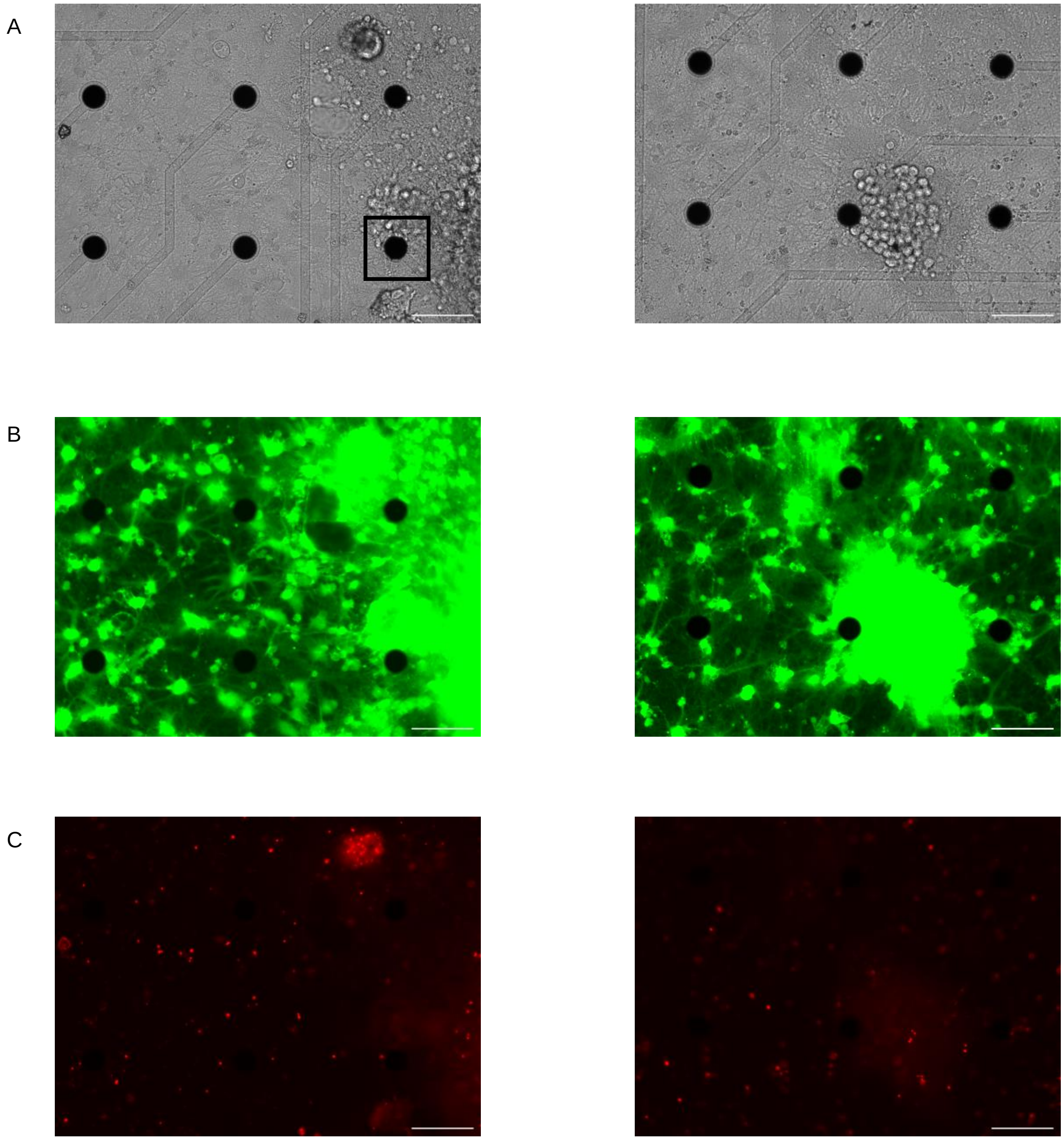

Figure S6. Live-dead assay of neuronal rat cells at the end of a longitudinal experiment. The concentration and incubation time for calcein and ethidium homodimer were chosen according to the recommendations of the manufacturer. (A) Bright-field image, enclosed in a square the area of interest which was used for running the integrated, sequential electrophysiological-spectroscopic analysis. (B) Calcein-image indicating live cells. (C) Ethidium-homodimer-image indicating dead cells. Scale bar size: 125 μm. Scale bar intensity: 0-4095 A.U.
